# Venetoclax and Azacitidine in Chinese patients with untreated acute myeloid leukemia ineligible for intensive chemotherapy

**DOI:** 10.1038/s41392-023-01394-8

**Published:** 2023-05-03

**Authors:** Leiming Xia, Wanlu Tian, Yiming Zhao, Lingling Jiang, Wei Qian, Lei Jiang, Ling Ge, Jianjun Li, Fengbo Jin, Mingzhen Yang

**Affiliations:** 1 Department of Hematology, the first affiliated hospital of Anhui Medical University, Hefei, Anhui 230022 China; 2 Anhui Public Health Clinical Center, Hefei, Anhui 230031 China; 3 Anhui Provincial Institute of Translational Medicine, Hefei, Anhui 230092 China

**Keywords:** Haematological cancer, Outcomes research

**Dear Editor**,

Acute Myeloid Leukemia (AML) patients are often found ineligible for aggressive standard chemotherapy in clinic because of old age and poor conditions. Current evidences suggest that targeted combination therapy might help these patients achieve a higher response rate with acceptable adverse events (AEs).^1^ Venetoclax (VEN), a selective small-molecule BCL2 inhibitor, combined with hypomethylating agent (HMA), such as azacytidine (AZA), is now the standard treatment for older patients ineligible for intensive chemotherapy.^2^ This combination therapy has proven to be effective in challenging AML population.^3^ Recently, a subgroup analysis in VIALE-A trial^1^ indicates that Chinese patients might not benefit more from a VEN plus AZA regimen compared with AZA alone. Unlike most other geographically defined populations in this same study, the patient number included in VIALE-A trial for Chinese population is relatively small. Due to a large population base with increasing AML patients in China, it would be important to further evaluate the effectiveness of such combo treatment in Chinese AML patients. Following a similar clinical design as the VIALE-A trial, this observational study was conducted to evaluate the efficacy and safety of VEN plus AZA combo compared with AZA alone in previously untreated Chinese AML patients who were ineligible for intensive induction chemotherapy. Further evidence was provided for associated genomic characteristics of Chinese AML patients who might benefit from such a combo treatment.

A total of 35 eligible patients was enrolled in this two-arm, single-center, randomized observational clinical trial (Chinese Clinical Trial Registry number: ChiCTR2200065106), with a 3 to 2 randomization—21 patients assigned to the VEN plus AZA group (referred to as the combo group) and 14 patients assigned to the placebo plus AZA group (referred to as the control group). The trial has been approved by the ethics committee of the first affiliated hospital of Anhui Medical University, and performed according to the Ethics Review on Biomedical Research Involving Human Subjects and the Declaration of Helsinki. Main inclusion criteria include a confirmed diagnosis of previously untreated AML according to the definition by World Health Organization, and ineligibility for standard chemo-induction therapy. The ineligibility for standard chemo-therapy was determined if the patient matches at least one of the following: (1) 75 years of age or older; (2) one or more of pre-existing conditions including heart failure, chronic stable angina, lung function insufficiency, invasive pulmonary fungal infection and serious infection; (3) an Eastern Cooperative Oncology Group (ECOG) performance-status score of 2 or 3. Main exclusion criteria of the trial is any previous treatment of hypomethylating agent, venetoclax, chemotherapy for myelodysplastic syndrome.

The baseline and clinical characteristics were examined and analyzed between combo and control groups, and found to be largely comparable, as summarized in supplementary Table 1. Patients in the combo group were orally administered in a treatment cycle of 28-days of VEN, with a regimen of q.d. of 100 mg on day 1, 200 mg on day 2, 400 mg on day 3 and continued at 400 mg daily until day 28, while patients in the control group received placebo orally following the same schedule. AZA was administered in both groups at a dose of 75 mg/m^2^ subcutaneously for the first 7 days of each treatment cycle. Such a treatment regimen was strictly followed and continued until disease progression, withdrawal of consent or other discontinuation circumstances as defined by the protocol.

In the follow-up study of 44 months, we evaluated and compared the efficacy of mono and combo treatment. The combo group exhibited an increased Overall Survival (OS) compared to the control group (hazard ratio, 0.5025; 95% CI, 0.2253 to 1.121; log-rank *p* = 0.0384). Specifically, median OS was 11.0 months (95% confidence interval [CI], 2–23) and 2.0 months (95% CI, 1–8) in the combo and control group, respectively; Progression Free Survival (PFS) with the combo group showed an upward trend compared to the control group, although statistically significant difference was not observed. Specifically, median PFS was 10.0 months (95% CI, 2–21) and 2 months (95% CI, 1–8) in the combo and control group (hazard ratio, 0.5542; 95% CI, 0.2524–1.217; log-rank *p* = 0.0819) (Fig. 1a). Furthermore, ORR and CR rates of best curative effect were significantly higher in the combo group (Fig. 1b*)*, with objective response rates (ORR, complete remission (CR) + partial remission (PR)) at 57.14% and 35.71% (*p* = 0.0223), and CR at 52.38% and 14.29% (*p* = 0.0480), in the combo and control group, respectively.

**Fig. 1:**
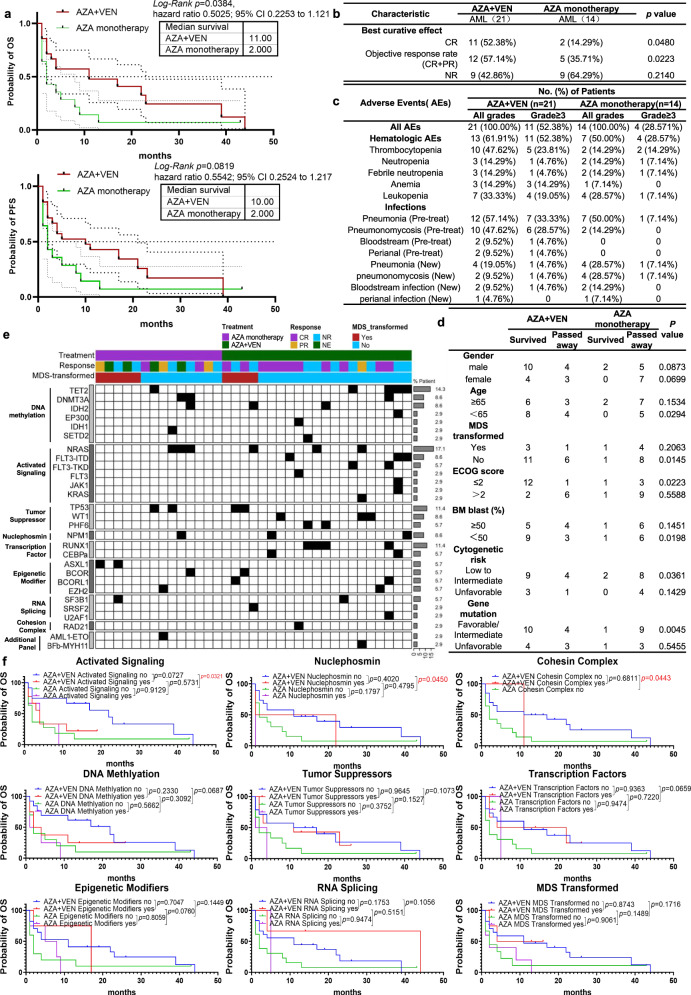
VEN plus AZA combo significantly improves the outcome of Chinese AML patients. **a** VEN plus AZA enhanced the OS of Chinese AML patients. A trend of increase in PFS was also observed. **b** VEN plus AZA combo significantly improves ORR compared with control group. **c** AEs were reported in all 35 patients. The hematologic AEs of grade 3 or higher included thrombocytopenia, neutropenia, febrile neutropenia, anemia, and leukopenia. Newly occured infection of grade 3 or higher included pneumonia, pneumonomycosis and bloodstream infection. No newly ocurred perianal infection of grade 3 or higher were observed in either group. **d** Subgroup Analysis of OS shows the Chinese AML patients with age <65 years, Non-MDS transformed, ECOG score ≤ 2, bone marrow blast <50%, low to intermediate risk of cytogenetic and low to intermediate risk of gene mutation were associated with higher survival rate (lower mortality rate) when treated with VEN plus AZA combo. **e** AML patients with specific gene mutations were grouped into nine functional signaling pathways, and analyzed for the treatment response to combo and control treatment. **f** OS was significantly longer in combo treatment compared with control with three pathways, e.g., activated signaling, nuclephosmin and cohesin complex harboring no mutations

These data suggests that previously untreated Chinese AML patients ineligible for intensive chemotherapy may benefit from a VEN plus AZA combo therapy. In contrast to the VIALE-A trial data showing CR in Chinese AML patients with VEN plus AZA therapy is lower than that in Japanese and American patients, suggesting this combo treatment may not benefit Chinese AML patients,^1^ our data demonstrates clearly an improved OS, ORR and CR in combo treatment compared to mono-treatment. Such a difference may be explained by the property of VIALE-A trial as a pan-geographic study with limited Chinese AML cohort scattered over several countries and clinical centers, and our study was designed to focus solely on Chinese AML patients with an increase of statistical power and narrowed confidence interval, where recruited patients received the same level of clinical care, support and treatment in a single-center clinical trial. Consistent with our study, a recent clinical trial showed that OS, but not PFS, was significantly improved in refractory/relapsed Chinese AML patients,^4^ when comparing VEN plus AZA combo to AZA only treatment.

Safety analysis showed that all patients suffered at least one AE. 11 patients in the combo (52.38%) and 4 in the control group (29.57%) experienced serious AEs (Grade 3 or higher) as shown in Fig. 1c. The most common AEs in the combo group are hematologic, with hematologic AEs of any grade occurring in 61.91% of patients in the combo group, compared to 50.00% in the control group. Frequency of serious hematologic AEs was 52.38% and 28.57% in the combo and control group, respectively. AEs of new infection during treatment were common and predominantly pneumonia (19.05% combo and 28.57% control), pneumonomycosis (9.52% combo and 28.57% control), bloodstream infection (9.52% combo and 14.29% control), perianal infection (4.76% combo and 7.14% control). Based on these data, the safety profile of VEN + AZA in Chinese AML patients observed in our study was consistent with the VIALE-A trial,^1^ with the exception that the incidence of grade 3 or higher hematologic AEs in Chinese AML patients appears to be lower than those in the overall population of VIALE-A trial^1^ (combo treatment: 52% current study vs 82% VIALE-A, control treatment: 29% current study vs 62% VIALE-A), which may reflect a differential AEs response among geographical populations.

In order to identify AML patients who might benefit from VEN plus AZA combo therapy, we classified and analyzed key clinical characteristics associated with clinical improvement. Patient’s mortality rate was compared between combo and control group for each category of clinical characteristics four months after the initial treatment. As shown in Fig. 1d, Chinese AML patients with an age <65 years, Non-MDS transformed, ECOG score ≤ 2, bone marrow blast <50%, low to intermediate cytogenetic risk and low to intermediate gene mutation risk were significantly associated with a higher survival rate (e.g. lower mortality rate) in the combo group compared with the control group.

In order to identify potential biomarkers associated with an enhanced efficacy through combo treatment in Chinese AML patients, we evaluated the association between specific gene mutations and patient OS. Genomic samples from bone marrow blasts collected from all 35 patients enrolled were sequenced for a panel of 47 AML-associated genes. Mutations were classified into nine functional pathways, including DNA methylation, activated signaling, tumor suppressor, nuclephosmin, transcription factors, epigenetic modifiers, RNA splicing, cohesin complex and additional panel containing two gene-fusion mutations (Fig. 1e). Statistical analysis revealed that an increase in OS in the combo group is significantly associated with three pathways, e.g., activated signaling, nuclephosmin and cohesin complex, when genes from these pathways harbor no mutations (Fig. 1f). ORR and NR was compared between groups, and no significant difference was observed (Supplementary Table 2). This result suggests that Chinese treatment naive AML patients without gene mutations in functional pathways of activated signaling, nuclephosmin and cohesin complex may benefit from the combo treatment.

Consistent with our finding that specific gene mutations may affect the treatment outcome of AZA plus VEN combo, emerging evidence shows that reconstructed existing mutations, especially dominant mutations, contributed to VEN resistance in AML.^5^ To potentiate this finding, we report two cases with a treatment regimen combining AZA plus VEN with an additional mutation-targeting drug, both of which achieved a satisfactory disease remission (Supplementary Fig. 1). The first case is a 43-year-old untreated AML male patient who achieved CR after initial induction chemotherapy of AZA plus VEN, and relapsed in the 12th month after maintenance treatment. Sequencing revealed multiple gene mutations with persistent FLT3-ITD mutation and additional mutations in genes including ARID2, ASXL3, ATM, CREBBP, MYBBP1A, PBRM1, PML, AMARCA2, TET1, and UNCL3D. Non-remission (NR) was observed after the first cycle of FLT3-ITD targeting drug, Sorafenib, plus VEN + AZA combo, while CR was achieved using a regimen of FLT3-ITD targeting Gilteritinib plus VEN + AZA combo in the second cycle, with persistent mutations of FLT3-ITD and CEBPA (Supplementary Fig. 1). The second case is a 72-year-old female with primary AML, with gene mutations of FLT3-ITD, NPM1, DNMT3A as sequenced immediately after diagnosis. This patient reached CR and molecular remission of FLT3-ITD after one cycle treatment of Sorafenib, plus VEN + AZA combo. These two clinical cases suggest that additional molecular targeting drugs may benefit AML patients with specific mutations, when the combo treatment results in resistance or relapse. As a result, specific gene mutations such as FLT3-ITD might negatively affect the treatment outcome of VEN plus AZA combo therapy, and mutation targeting drugs in a personalized setting, based on sequencing, might confer a response and improved efficacy to VEN + AZA combo in patients with specific mutations.

Taken together, by focusing on the efficacy, safety and genomic characteristics of VEN plus AZA combo treatment, our study demonstrated for the first time that VEN plus AZA combo treatment may significantly improve the clinical outcome in previously untreated Chinese AML patients. For patients harboring specific gene mutations such as FLT3-ITD, molecular targeting drugs, such as Sorafanib and Gilteritinib, may further help improve the clinical outcome of VEN plus AZA combo in a more personalized setting. Limitations of this study remains as it is a single-center study with a relatively small cohort, which with its findings would warrant further study to further confirm such a beneficial effect with combo therapy in treatment-naïve Chinese AML patients.

## Supplementary information

Supplementry Material

## Data Availability

All data are available from the corresponding author. The gene sequencing data were derived from patients’ clinical reports.
