# Supplementary material for: Venetoclax and Azacitidine in Chinese patients with untreated acute myeloid leukemia ineligible for intensive chemotherapy
Source: Signal Transduct Target Ther. 2023 May 3;8:176. doi: 10.1038/s41392-023-01394-8 (PMC10154410; doi:10.1038/s41392-023-01394-8)
Supplement: Supplementary file 1 — Supplementry Material [file 41392_2023_1394_MOESM1_ESM.docx]

Supplementary Materials for

# Venetoclax and Azacitidine in Chinese patients with untreated acute myeloid leukemia ineligible for intensive chemotherapy

Leiming Xia^1, 2, 3, *, #^, Wanlu Tian^1, 2, *^, Yiming Zhao^1, 2, *^, Lingling Jiang^1, 2, *^, Wei Qian^1, 2^, Lei Jiang^1, 2^, Ling Ge^1, 2^, Jianjun Li^1, 2^, Fengbo Jin^1, 2, #^, Mingzhen Yang^1, 2, #^

Correspondence to: Leiming Xia ([278461175@qq.com](mailto:278461175@qq.com)), Fengbo Jin ([13855191690@163.com](mailto:13855191690@163.com)), Mingzhen Yang ([yangmz89@163.com](mailto:yangmz89@163.com))

**This PDF file includes:**

Materials and Methods

Figures S1

Tables S1 to S2

Materials and Methods

**Patients**

A total of 35 eligible patients was enrolled in this two-arm, single-center, randomized observational clinical trial (Chinese Clinical Trial Registry number: ChiCTR2200065106), with a 3 to 2 randomization - 21 patients assigned to the Venetoclax (VEN) and Azacitidine (AZA) group (referred to as the combo group) and 14 patients assigned to the AZA plus placebo group (referred to as the control group). Main inclusion criteria include a confirmed diagnosis of previously untreated AML according to the definition by World Health Organization, and ineligibility for standard chemo-induction therapy. The ineligibility for standard chemo-therapy was determined if the patient matches at least one of the following: 1) 75 years of age or older; 2) One or more of pre-existing conditions including heart failure, chronic stable angina, lung function insufficiency, invasive pulmonary fungal infection and serious infection; 3) an Eastern Cooperative Oncology Group (ECOG) performance-status score of 2 or 3. Main exclusion criteria of the trial is any previous treatment of hypomethylating agent, venetoclax, chemotherapy for myelodysplastic syndrome. The diagnosis of AML for all patients were confirmed at the first Affiliated Hospital of Anhui Medical University between January 2019 and December 2021, and fellow-up to December 2022.

**Ethics statement**

The trial has been approved by the ethics committee of the first affiliated hospital of Anhui Medical University, and performed according to the Ethics Review on Biomedical Research Involving Human Subjects and the Declaration of Helsinki. Patients’ data were handled and deidentified according to ethical and legal standards. Written informed consent were obtained from all participants in the study.

**Trial Design and Regimens**

Eligible patients were randomly assigned to VEN plus AZA group (AZA+VEN) or AZA plus placebo group (AZA monotherapy) in a 3:2 ratio. Patients in the combo group were orally administered in a treatment cycle of 28-days of VEN, with a regimen of q.d. of 100 mg on day 1, 200 mg on day 2, 400 mg on day 3 and continued at 400mg daily until day 28, while patients in the control group received placebo orally following the same schedule. AZA was administered in both groups at a dose of 75 mg/m^2^ subcutaneously for the first 7 days of each treatment cycle. Such a treatment regimen was strictly followed and continued until disease progression, withdrawal of consent or other discontinuation circumstances as defined by the protocol.

**End Points and** **Assessments**

The primary end points were OS and progression-free survival (PFS). The secondary end points were CR and partial remission (PR). OS was defined as the number of months from randomization to the date of death; PFS was defined as the number of months from randomization to disease progression, confirmed disease relapse, or death. CR was defined as normal blood corpuscle counting, blood transfusion independence, and bone marrow with less than 5% blasts. PR was defined as 5-25% blasts in the bone marrow and a total reduction of blasts of at least 50% of AML blasts. All patients who received at least one dose of either AZA or VEN were included in the safety analysis. Treatment-related adverse events were defined as those that occurred from the first dose until 30 days after the discontinuation of treatment. The severity of adverse events was graded according to the National Cancer Institute Common Terminology Criteria for Adverse Events, version 4.03. Cytogenetic risk and genetic alterations were evaluated according to the NCCN guidelines for AML, version 2.2021.

**Statistical Analysis**

The data cutoff date was 31^st^ October, 2022. The baseline characteristics were descriptive. The efficacy analysis was performed using intent-to-treat population. The distribution of OS was estimated for each treatment group using Kaplan–Meier method and compared with the use of the log-rank test. The hazard ratio between the treatment groups was estimated with the Cox proportional-hazards model. CR and PR were compared between the treatment groups with the use of the Pearson’s Chi-square test.

**Figure S1.**

**Fig. S1** Two cases with a treatment regimen combining AZA+VEN with an additional mutation-targeting drug achieved a satisfactory disease remission. The 1^st^ case is a 43 years old untreated AML male patient who achieved CR after initial induction chemotherapy of AZA plus VEN, and relapsed in the 12^th^ month after maintenance treatment. Sequencing revealed multiple gene mutations with persistent FLT3-ITD mutation and additional mutations in genes including ARID2, ASXL3, ATM, CREBBP, MYBBP1A, PBRM1, PML, AMARCA2, TET1 and UNCL3D. Non-Remission (NR) was observed after the first cycle of FLT3-ITD targeting drug, Sorafenib, plus VEN + AZA combo, while CR was achieved using a regimen of FLT3-ITD targeting Gilteritinib plus VEN + AZA combo in the second cycle, with persistent mutations of FLT3-ITD and CEBPA. The 2^nd^ case is a 72 years old female with primary AML, with gene mutations of FLT3-ITD, NPM1, DNMT3A as sequenced immediately after diagnosis. This patient reached CR and molecular remission of FLT3-ITD after one cycle treatment of Sorafenib, plus VEN + AZA combo.

**Table S1** Baseline Demographic and Clinical Characteristics of the Patients.

﻿

| **Characteristic** | **AZA+VEN** | **AZA monotherapy** | ***p* value** |
| --- | --- | --- | --- |
|  | **AML（21）** | **AML（14）** |  |
| **Age** |  |  | 0.1415 |
| Median (range) | 63(24-77) | 73(17-80) |  |
| **Male sex no. (%)** | 14(66.67%) | 7(50%) | 0.4830 |
| **AML type no (%)** |  |  | 0.4318 |
| De novo | 17(80.95%) | 9(64.29%) |  |
| MDS transformed AML | 4(19.05%) | 5(35.71%) |  |
| **ECOG score** |  |  | 0.2543 |
| 1 | 2 | 1 |  |
| 2 | 11 | 3 |  |
| 3 | 4 | 6 |  |
| 4 | 4 | 4 |  |
| **Bone Marrow Blast (%)** | |  | 0.7210 |
| ≤30 | 6 | 5 |  |
| ＞30 | 15 | 9 |  |
| **Cytogenetic risk** |  |  | 0.3616 |
| Favorable | 2 | 2 |  |
| Intermediate | 11 | 8 |  |
| Unfavorable | 4 | 4 |  |
| Unknown | 4 | 0 |  |
| **Gene mutation** |  |  | 0.4888 |
| FLT3 ITD or TKD | 6 | 0 |  |
| NPM1 | 2 | 1 |  |
| TP53 | 2 | 2 |  |
| ASXL1 | 0 | 2 |  |
| **background disease no.** | |  | 0.4823 |
| 1 | 4 | 1 |  |
| 2 | 10 | 6 |  |
| 3-4 | 7 | 7 |  |

**Table S2** ORR and NR comparison for AZA+VEN and AZA only groups based on nine functional pathways of gene mutations.

|  | **AZA+VEN** | | **AZA monotherapy** | | ***p* value** |
| --- | --- | --- | --- | --- | --- |
|  | **CR+PR** | **NR** | **CR+PR** | **NR** |  |
| **DNA Methlyation** |  |  |  |  |  |
| negative | 5 | 3 | 9 | 4 | >0.9999 |
| 1-2 | 0 | 1 | 2 | 4 | >0.9999 |
| ≥2 | 0 | 1 | 1 | 1 | >0.9999 |
| **Activated Signaling** |  |  |  |  |  |
| negative | 5 | 3 | 8 | 4 | >0.9999 |
| 1-2 | 0 | 2 | 3 | 4 | 0.5000 |
| ≥2 | 0 | 0 | 1 | 1 | >0.9999 |
| **Tumor Suppressors** |  |  |  |  |  |
| negative | 5 | 4 | 8 | 6 | >0.9999 |
| 1-2 | 0 | 1 | 4 | 3 | >0.9999 |
| **Nuclephosmin** |  |  |  |  |  |
| negative | 5 | 5 | 11 | 8 | 0.7141 |
| positive | 0 | 0 | 1 | 1 | >0.9999 |
| **Transcription Factors** |  |  |  |  |  |
| negative | 5 | 5 | 9 | 6 | 0.6968 |
| positive | 0 | 0 | 3 | 3 | >0.9999 |
| **Epigenetic Modifiers** |  |  |  |  |  |
| negative | 4 | 3 | 9 | 8 | >0.9999 |
| 1-2 | 1 | 2 | 3 | 1 | 0.4857 |
| **RNA Splicing** |  |  |  |  |  |
| negative | 5 | 4 | 10 | 8 | >0.9999 |
| 1-2 | 0 | 1 | 2 | 1 | >0.9999 |
| **Cohesin Complex** |  |  |  |  |  |
| negative | 5 | 5 | 11 | 9 | >0.9999 |
| positive | 0 | 0 | 1 | 0 | >0.9999 |
| **Pathways involved based on gene mutation** |  |  |  |  |  |
| ＜2 | 4 | 2 | 6 | 2 | >0.9999 |
| ≥2 | 1 | 3 | 6 | 7 | 0.6029 |
| **MDS transformed** |  |  |  |  |  |
| Yes | 1 | 2 | 2 | 2 | >0.9999 |
| NO | 4 | 3 | 10 | 7 | >0.9999 |
